# Phantom study and clinical application of total-body ^18^F-FDG PET/CT imaging: How to use small voxel imaging better?

**DOI:** 10.1186/s40658-023-00597-w

**Published:** 2024-02-15

**Authors:** Chi Qi, Xiuli Sui, Haojun Yu, Siyang Wang, Yan Hu, Hongyan Sun, Xinlan Yang, Yihan Wang, Yun Zhou, Hongcheng Shi

**Affiliations:** 1 Department of Nuclear Medicine, Zhongshan Hospital, Fudan University, No. 180 in Fenglin Road, Shanghai, 200032 People’s Republic of China; 2 Institute of Nuclear Medicine, Fudan University, Shanghai, 200032 People’s Republic of China; 3 Shanghai Institute of Medical Imaging, Shanghai, 200032 People’s Republic of China; 4 Cancer Prevention and Treatment Center, Zhongshan Hospital, Fudan University, Shanghai, 200032 People’s Republic of China; 5 Department of Nuclear Medicine, The Affiliated Hospital of Southwest Medical University, Luzhou, Sichuan 646000 People’s Republic of China; 6 Central Research Institute, United Imaging Healthcare Group Co, Ltd, Shanghai, People’s Republic of China

**Keywords:** Long-axial PET/CT, Large matrix, Small voxel, Small lesion detection

## Abstract

**Background:**

Conventional PET/CT imaging reconstruction is typically performed using voxel size of 3.0–4.0 mm in three axes. It is hypothesized that a smaller voxel sizes could improve the accuracy of small lesion detection. This study aims to explore the advantages and conditions of small voxel imaging on clinical application.

**Methods:**

Both NEMA IQ phantom and 30 patients with an injected dose of 3.7 MBq/kg were scanned using a total-body PET/CT (uEXPLORER). Images were reconstructed using matrices of 192 × 192, 512 × 512, and 1024 × 1024 with scanning duration of 3 min, 5 min, 8 min, and 10 min, respectively.

**Results:**

In the phantom study, the contrast recovery coefficient reached the maximum in matrix group of 512 × 512, and background variability increased as voxel size decreased. In the clinical study, SUV_max_, SD, and TLR increased, while SNR decreased as the voxel size decreased. When the scanning duration increased, SNR increased, while SUV_max_, SD, and TLR decreased. The SUV_mean_ was more reluctant to the changes in imaging matrix and scanning duration. The mean subjective scores for all 512 × 512 groups and 1024 × 1024 groups (scanning duration ≥ 8 min) were over three points. One false-positive lesion was found in groups of 512 × 512 with scanning duration of 3 min, 1024 × 1024 with 3 min and 5 min, respectively. Meanwhile, the false-negative lesions found in group of 192 × 192 with duration of 3 min and 5 min, 512 × 512 with 3 min and 1024 × 1024 with 3 min and 5 min were 5, 4, 1, 4, and 1, respectively. The reconstruction time and storage space occupation were significantly increased as the imaging matrix increased.

**Conclusions:**

PET/CT imaging with smaller voxel can improve SUV_max_ and TLR of lesions, which is advantageous for the diagnosis of small or hypometabolic lesions if with sufficient counts. With an ^18^F-FDG injection dose of 3.7 MBq/kg, uEXPLORER PET/CT imaging using matrix of 512 × 512 with 5 min or 1024 × 1024 with 8 min can meet the image requirements for clinical use.

**Supplementary Information:**

The online version contains supplementary material available at 10.1186/s40658-023-00597-w.

## Background

Positron emission tomography/computed tomography (PET/CT) can provide both morphological features and metabolic functional information simultaneously, which is widely used for tumor diagnosis, staging, restaging, treatment evaluation, etc. [1, 2].

Diagnostic accuracy in PET/CT highly depends on the image quality, including the parameters like signal-to-noise ratio (SNR), spatial resolution, partial volume effect (PVE), and target-to-nontarget ratio (T/NT). Images of different sizes will significantly affect the image quality in clinical application; thus, the aforementioned parameters need to be balanced for a better image quality. However, limited by the intrinsic physical and technical shortages such as short physical coverage, the low intrinsic spatial resolution and photon detection efficiency make it difficult to detect small lesions using a large imaging matrix in PET imaging [3]. Nowadays, many methods in both hardware and software have been applied to improve image quality, such as longer axis scanners, new scintillation crystals, new photomultiplier, time-of-flight (TOF) [4], point spread function (PSF) [5], and new reconstruction algorithms [6, 7].

The conventional clinical PET scanners typically have an axial field-of-view (AFOV) of 15–30 cm, resulting in limited coverage and relatively low photon detection efficiency [8]. Limited by the low system sensitivity, the conventional PET/CT image reconstruction is traditionally performed using a voxel size in a range of 3.0–4.0 mm in the three perpendicular directions [9]. Typically a larger voxel size results in a lower spatial resolution of the reconstructed PET image, which limits the display of small lesions, especially those with low metabolism uptake [10]. A smaller voxel size PET imaging has demonstrated the potential advantages of improved image quality and diagnosis accuracy in a number of preclinical and clinical studies [11–13]. A few studies have evaluated the use of small voxel imaging such as 2 × 2 × 2 mm^3^ in conventional PET/CT to improve signal-to-noise ratios in a phantom study, demonstrating the potential of small voxel imaging in improving the accuracy of small lesion detection [14, 15]. Taking advantages of the development of the long-axial PET/CT technology, the uEXPLORER PET/CT scanner (United Imaging Healthcare, Shanghai, China) with an axial coverage of 194 cm increases the effective count rate by about 40 times than that of conventional PET scanners [16, 17], leading to tremendous progress in system sensitivity. Previous studies have proved that uEXPLORER PET/CT can shorten acquisition time or reduce the injected dose while maintaining comparable image quality and lesion detectability relative to the conventional short-axis PET/CT in oncological studies [18, 19]. The high sensitivity of uEXPLORER has allowed the investigation of exploring the diagnosis accuracy of small lesion by using small voxel imaging.

So far, it is not clear whether the total-body PET/CT imaging with a smaller voxel size (1.2 × 1.2 × 1.4 mm^3^ or even smaller as 0.6 × 0.6 × 1.4 mm^3^) could be applicable for clinical use with the same acquisition time and radiotracer dose condition as that the conventional PET/CT does.

In this study, we will explore the benefits of detecting and evaluating small or hypometabolic lesions brought by using the long-axis PET/CT with smaller voxel imaging and find the recommended clinical conditions based on both phantom and clinical studies.

## Materials and methods

### Phantom and clinical acquisition protocols

For the phantom study, the National Electrical Manufacturers Association (NEMA) NU-2–2018 image quality (IQ) phantom [20] was evaluated. All spheres were filled with an ^18^F-FDG solution at a sphere-to-background concentration ratio of 4:1 and with a background activity concentration of 5.3 kBq/ml. The IQ phantom was positioned with the spheres at the axial center of the PET FOV. A single-bed position was used with a scanning duration of 30 min.

This clinical study was approved by the Institutional Review Board of Zhongshan Hospital, Fudan University. Thirty colorectal patients who underwent total-body ^18^F-FDG PET/CT for cancer diagnosis, staging, or restaging were retrospectively enrolled in this study, and the basic information about the patient and the lesions should be found in Table 1. The patients were refrained from strenuous exercise within 24 h and fasted for 6 h before the injection of ^18^F-FDG with a dose of 3.7 MBq/kg. The blood glucose level should be less than 11 mmol/l according to the guideline of the European Association of Nuclear Medicine for FDG PET/CT oncological examinations [9]. All patients rested quietly for about 60 min after injection of ^18^F-FDG, and then total-body PET/CT scan was performed to acquire from the top of the skull to the soles of the feet for 15 min.

**Table 1:** The basic information about patients and lesions

Characteristic	Value
Age (years)	55.8 ± 12.7 (32–79)
Sex	30
Male	17 (56.7%)
Female	13 (43.3%)
BMI (kg/m^2^)	23.1 ± 3.3 (17.3–32.9)
Blood glucose before injection (mmol/L)	5.7 ± 1.3
Injected dose (MBq)	28.5 ± 6.7 (20.3–53.0)
Acquisition time (min)	15
Primary site of colorectal cancer	
Colon	20
Rectum	10
Number of metastases	129
Size of metastases (mm)	
≤ 7	35
> 7 and ≤ 15	76
> 15 and ≤ 25	18

Both the phantom and patients were scanned in a list-mode using the uEXPLORER PET/CT scanner. Low-dose CT imaging is performed before PET acquisition for attenuation correction and positioning. The CT scanning parameters were set as follows: tube voltage 120 kV, tube current 140 mAs, and pitch 1.0.

### PET/CT image reconstruction

All the PET images were reconstructed using the ordered subset expectation maximization (OSEM) algorithm with TOF and PSF (OSEM-TOF-PSF) applied. For the phantom study, two iterations, 20 subsets, 1.4 mm slice thickness, and 600 mm FOV with a Gaussian post-filter of 3 mm were employed. For the clinical study, three iterations, 20 subsets, slice thickness of 1.4 mm, and FOV of 600 mm with a Gaussian post-filter of 3 mm were used to be consistent with the clinical application requirements. To make the large-matrix imaging reconstruction suitable for the routine clinical imaging needs, only images from the base of the skull to the root of the thigh were reconstructed. The images were corrected for radioactive decay, attenuation, scatter, random, and dead time. In the following context, each reconstruction group will be expressed as G192-3, representing the reconstruction imaging matrix of 192 by 192 with a scanning duration of 3 min. To roughly assess the effect of the matrix on the SNR and to determine the combinations of image matrices and scanning durations, G192-3, G512-3, and G1024-3 were pre-reconstructed and the combinations selected in this study are shown in Table 2.

**Table 2:** The combination of image matrices and scanning durations reconstructed for phantom and clinical study

Type	Image matrix	Voxel size (mm^3^)	Scanning duration (min)
Phantom	192 × 192	3.1 × 3.1 × 2.9	3; 5
	512 × 512	1.2 × 1.2 × 2.9	3; 5; 8; 10
	1024 × 1024	0.6 × 0.6 × 2.9	
Clinical Patient	192 × 192	3.1 × 3.1 × 1.4	3; 5
	512 × 512	1.2 × 1.2 × 1.4	3; 5; 8; 10
	1024 × 1024	0.6 × 0.6 × 1.4	

### Image evaluation for phantom study

The time frames from the original 30-min duration were truncated to simulate shorter scanning durations, i.e., 3 min, 5 min, 8 min, and 10 min. The contrast recovery coefficient (CRC) and background variability were calculated to evaluate the image quality of the combinations with different image matrices and scanning durations.

The local contrast recovery coefficient (CRC_local_) was defined by

$${\mathrm{CRC}}_{\mathrm{local}}=\frac{\frac{H}{B}-1}{R-1}$$

where *H* is the average count within the region of interest (ROI) in the hot sphere,* B* is the average count in the annulus ROI around the spheres, and *R* is the experimental setup hot-to-background ratio.

For the cold source, CRC_local_ is defined by

$${\mathrm{CRC}}_{\mathrm{local}}=1-\frac{C}{B}$$

where *C* was the average count of the ROI in the cold sphere.

The background variability is defined as the standard deviation of the background ROI counts over *B*.

For evaluation, the CRC curve vs background variability was plotted for the combinations with different image matrices and scanning duration.

### Image evaluation for clinical Study

#### Objective analysis

The objective analysis of image quality was performed by two experienced nuclear radiologists. To evaluate the blood pool, a circular ROI was drawn as large as possible in the descending aorta at the bronchial bifurcation. Two ROIs with a diameter of 20 mm were drawn in the right lobe and the left external lobe of the liver at the portal vein bifurcation, and needed to avoid any lesions and large vessels. Two ROIs with a diameter of 20 mm were placed in the bilateral gluteus maximus at the largest cross-sectional area for assessing the muscle. All ROIs in the same organ should be kept in the same location and size in all the reconstruction groups for the same patient. The maximum standard uptake value (SUV_max_), the mean standard uptake value (SUV_mean_), and the standard deviation (SD) of the ROI were calculated. The SNR was calculated by dividing the SUV_mean_ by SD.

#### Subjective analysis

**Fig. 1:**
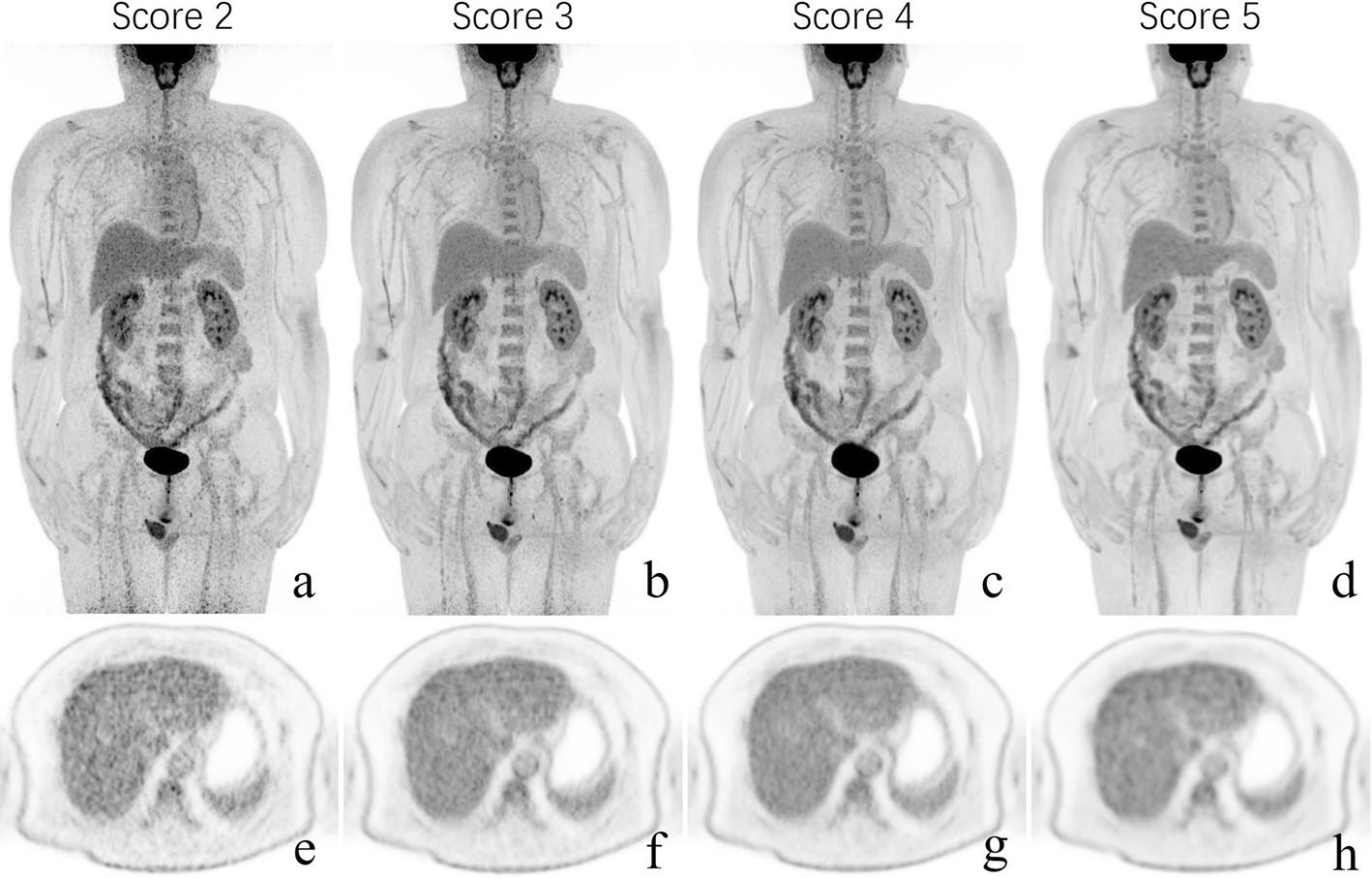
Description of the 5-point Likert scales for subjective assessment of the image quality. Description of the 5-point Likert score for qualitative assessment of the image quality (2–5 from the left to the right, respectively). Score 1, image with nondiagnostic quality; score 2, image which is acceptable, but with sub-optimal noise, lesion depiction leading to impaired diagnostic confidence; score 3, image with quality that is equivalent to routine clinical practice; score 4, image with quality that is superior to the average image quality; score 5, image with excellent quality

A 5-point Likert scale (5-PS) was used to subjectively evaluate image quality based on the following three perspectives: the overall impression, the image noise, and the conspicuity of the lesions, as illustrated in Fig. 1. Two experienced nuclear medicine physicians (with 5 and 9 years of experience in PET/CT diagnosis, respectively) were trained by reading 10 additional PET/CT cases together to understand the criteria before evaluation. The average of the mediastinal, hepatic, and pelvic in the maximum intensity projection (MIP) image was taken as the final score for each physician. The average of the two final scores was taken as the overall score for one reconstruction group.

#### Lesion detection

Lesions detection was carried out by two nuclear medicine physicians, and the final results were confirmed by MRI and pathological information. The SUV_max_ of the lesions was also measured by delineation of the volume of interest (VOI), and the size (maximum radial length) of the lesions was measured in conjunction with CT and MRI. For patients with multiple lesions, 3–5 hypometabolic lesions with a size smaller than 2.5 cm were selected. The lesion-to-liver ratio (TLR) was calculated by dividing the SUV_max_ of lesions by the SUV_mean_ of the liver in the same group.

### Reconstruction workstation configuration parameters

The configuration of the reconstruction workstation was as follows: two Intel Xeon 6126 CPUs with 2.6 GHz processor, 16 GB of DDR4 RAM, 1 TB HDD and two 2 TB SSD, and two TESLA PCI-E V100 GPUs with 24 GB memory.

### Statistical analysis

Statistical analyses were performed using the R software version 4.1.2 (R Core Team, 2022). Descriptive data were expressed as mean ± SD. The objective evaluation indexes were first applied the Shapiro–Wilk’s test to check the normality. Because of the non-normal distribution of the data, the nonparametric Freidman test was performed to compare the objective evaluation indexes among different scanning durations time within imaging matrices. When significant differences among groups were indicated by the Friedman test, the pairwise comparisons were conducted using a Wilcoxon signed-rank test. The p values were shown after Bonferroni correction, and a *p* value < 0.05 was considered statistically significant. The inter-rater agreement for subjective image quality score was analyzed via the Cohen’s kappa test, and Kappa values were interpreted as follows: 0.00–0.20, poor agreement; 0.21–0.40, fair agreement; 0.41–0.60, moderate agreement; 0.61–0.80, good agreement; and 0.80–1.00, almost perfect agreement.

### Image reconstruction time and storage space occupation

To examine the trade-offs between the clinical resources and the benefits brought by the proposed clinical protocols, the reconstruction time and the storage space occupation for each group were recorded.

## Results

### Phantom study

**Fig. 2:**
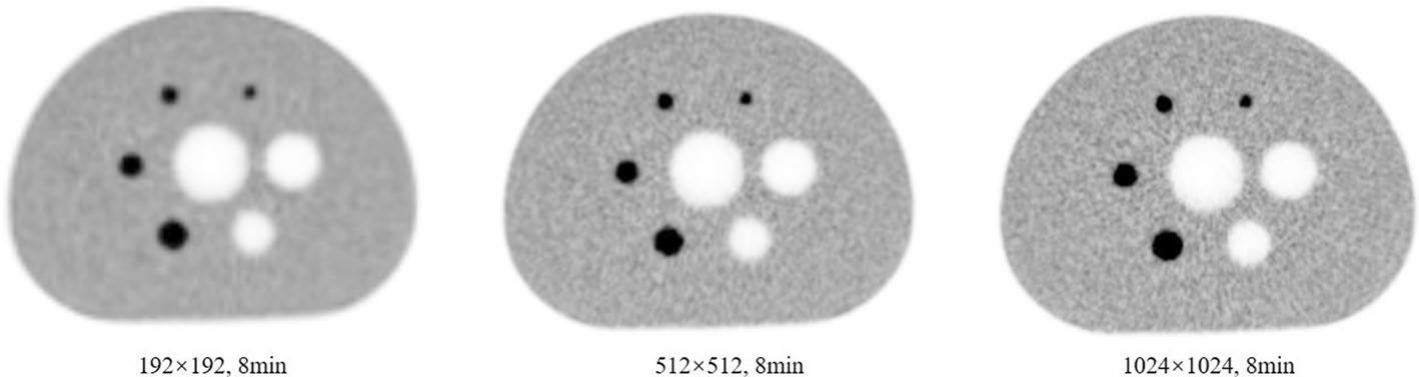
The images of the NEMA IQ phantom reconstructed with the different matrices with the scanning duration of 8 min

Figure 2 shows the images of the central slice of the NEMA IQ phantom reconstructed using different image matrices with a scanning duration of 8 min. As expected, PVE tends to be less obvious while the background variability increases as the image matrix increases.

**Fig. 3:**
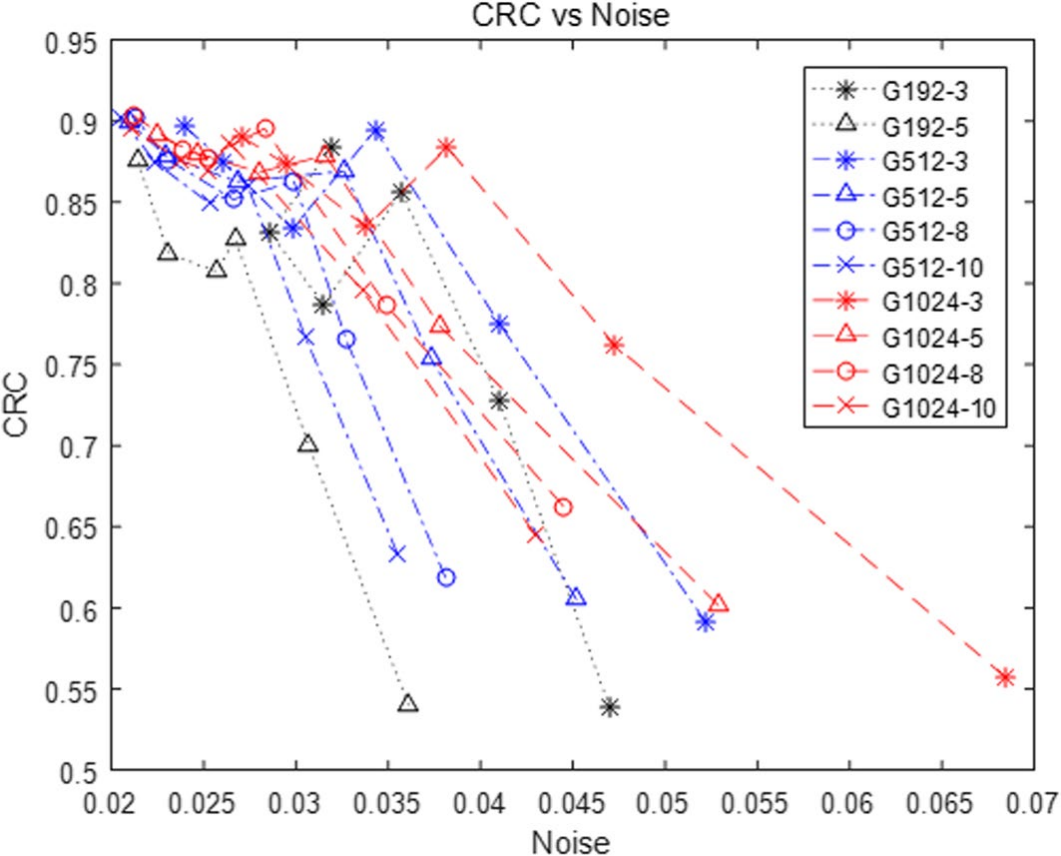
The contrast recovery coefficient vs the background variability for NEMA IQ phantom combine different image matrices with different scanning durations

To quantitatively illustrate the result, the CRC versus background variability for different matrices and scanning duration were plotted as shown in Fig. 3. The points on each curve from the left to right represent the spheres with diameters of 37, 28, 22, 17, 13, and 10 mm, respectively. For most cases as shown in this figure, CRC reached to the maximum with an imaging matrix of 512 by 512, and background variability increased as the voxel size became smaller. Considering the goal of this study, the parameters for the smallest sphere (diameter of 10 mm) are most relevant to the diagnosis accuracy of colorectal cancer metastasis. Therefore, for the 10 mm sphere, the CRC in G512-3 was increased by 5.2% and 3.4%, and the background variability was increased by 0.5%and 1.6% compared to G192-3 and G1024-3, respectively. The same trend can be found for the scanning duration of 5 min, 8 min, and 10 min.

### Clinical study

#### Objective assessment of image quality

**Fig. 4:**
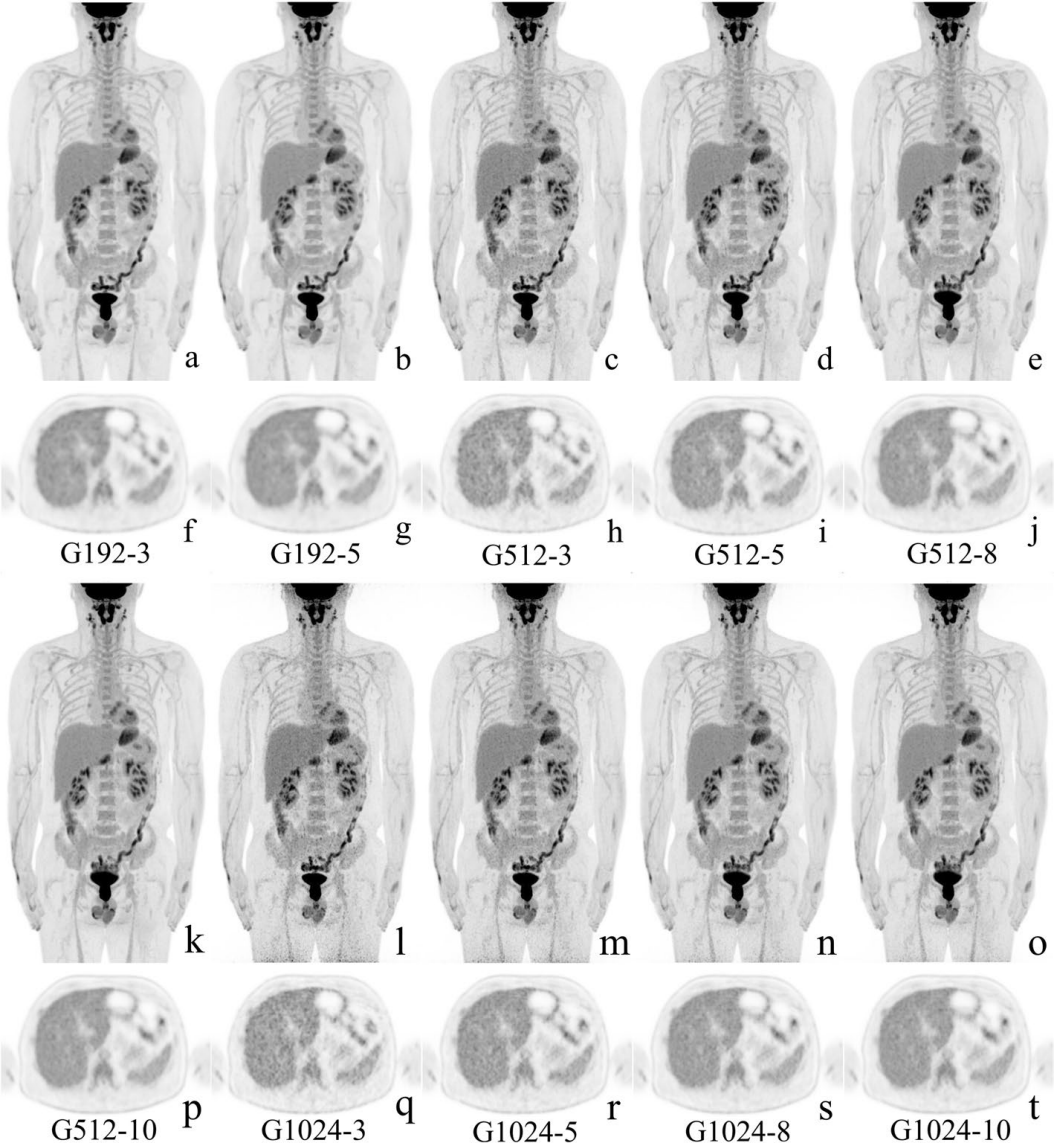
Typical MIP and cross-sectional images for different image matrices with different scanning durations. A 43-year-old male was diagnosed with rectum adenocarcinoma with parenteral lymph node metastasis. (**a**–**e**) and (**k**–**o**) showed the maximum intensity projection images for 10 different matrices and scanning durations, respectively. And **f**–**j** and **p**–**t** show cross-sectional images of the liver for the corresponding sequences

Figure 4 shows the typical images of a patient with 10 different reconstruction combinations. Background objective evaluation indexes in the liver among the 10 designed groups are summarized in Table 3, and more information regarding the mediastinal blood pool and the gluteus maximus can be found in Additional file 1: Table S1.

**Table 3:** The objective evaluation indexes for the combinations of the different imaging matrices and scanning durations

Index	G192-3	G192-5	G512-3	G512-5	G512-8	G512-10	G1024-3	G1024-5	G1024-8	G1024-10
SUV_max,liver_	2.67 ± 0.26	2.61 ± 0.25	2.91 ± 0.31	2.79 ± 0.28	2.70 ± 0.26	2.66 ± 0.26	3.09 ± 0.34	2.92 ± 0.29	2.80 ± 0.27	2.74 ± 0.27
SUV_mean,live_	2.39 ± 0.23	2.38 ± 0.23	2.38 ± 0.25	2.37 ± 0.24	2.35 ± 0.23	2.34 ± 0.23	2.40 ± 0.25	2.39 ± 0.24	2.37 ± 0.23	2.36 ± 0.23
SD_liver_	0.13 ± 0.04	0.12 ± 0.04	0.20 ± 0.04	0.17 ± 0.03	0.14 ± 0.03	0.13 ± 0.03	0.25 ± 0.05	0.20 ± 0.04	0.16 ± 0.03	0.15 ± 0.03
SNR_liver_	20.18 ± 5.9	22.43 ± 6.77	12.43 ± 2.51	14.66 ± 2.88	17.42 ± 3.61	18.68 ± 4.11	10.05 ± 1.86	12.34 ± 2.08	14.9 ± 2.46	16.46 ± 2.90
SUV_max,lesion_	5.94 ± 2.97	5.96 ± 2.96	6.43 ± 2.79	6.36 ± 2.73	6.25 ± 2.65	6.21 ± 2.65	6.63 ± 2.80	6.5 ± 2.69	6.34 ± 2.59	6.31 ± 2.60
TLR	2.47 ± 1.27	2.49 ± 1.28	2.67 ± 1.21	2.66 ± 1.20	2.65 ± 1.18	2.64 ± 1.19	2.73 ± 1.20	2.7 ± 1.17	2.67 ± 1.15	2.67 ± 1.16

*SUV*_*max,liver*_ SUV_max_ of liver, *SNR* signal-to-noise ratio, *SUV*_*max,lesion*_ SUV_max_ of lesion, *TLR* lesion-to-liver ratio

As shown in Table 3, the SUV_max_, SD, and TLR with the same scanning duration increased as the imaging matrix increased, and decreased as the scanning duration increased for the same imaging matrix groups. As shown in Table 4, the p values of SUV_max_ and SD from the Friedman test and post hoc tests were less than 0.05, except for the SUV_max,lesion_ compared between 8 and 10 min. The SUV_mean_ was close among all groups, although the Freidman test showed a significant difference, some of the pairwise comparisons were not statistically significant. The SNR increased as the scanning duration increased for the same imaging matrix group, and decreased as the voxel size turned smaller for the same scanning duration. All the comparisons of SNR, including the Friedman test and post-hoc pairwise tests, showed significantly different. These results indicated that a combination of a larger voxel size and a longer scanning duration can achieve lower SUV_max_, SD, and higher SNR.

**Table 4:** Comparisons of objective evaluation indexes of image quality among different scanning durations time within imaging matrices

Matrix	Index	*P*_*0*_	*P*_*1*_	*P*_*2*_	*P*_*3*_	*P*_*4*_	*P*_*5*_	*P*_*6*_
512×512	SUV_max,liver_	< 0.001	< 0.001	< 0.001	< 0.001	< 0.001	< 0.001	< 0.001
	SUV_mean,live_	< 0.001	0.155	0.031	0.005	0.074	0.005	0.002
	SD_liver_	< 0.001	< 0.001	< 0.001	< 0.001	< 0.001	< 0.001	< 0.001
	SNR_liver_	< 0.001	< 0.001	< 0.001	< 0.001	< 0.001	< 0.001	< 0.001
1024×1024	SUV_max,liver_	< 0.001	< 0.001	< 0.001	< 0.001	< 0.001	< 0.001	< 0.001
	SUV_mean,live_	0.001	0.676	0.130	0.037	0.147	0.011	0.010
	SD_liver_	< 0.001	< 0.001	< 0.001	< 0.001	< 0.001	< 0.001	< 0.001
	SNR_liver_	< 0.001	< 0.001	< 0.001	< 0.001	< 0.001	< 0.001	< 0.001
512×512	SUV_max,lesion_	< 0.001	0.017	< 0.001	< 0.001	0.003	0.004	0.249
	TLR	0.600	1.000	0.271	1.000	1.000	1.000	1.000
1024×1024	SUV_max,lesion_	< 0.001	< 0.001	< 0.001	< 0.001	< 0.001	< 0.001	0.926
	TLR	< 0.001	0.063	0.002	0.021	0.099	1.000	1.000

*P*_*0*_ indicates *P* values of Freidman test among four time groups; *P*_*1*_ to *P*_*6*_ indicates adjusted *P* values after Bonferroni corrections from comparisons between 3 and 5 min, between 3 and 8 min, between 3 and 10 min, between 5 and 8 min, between 5 and 10 min, and between 8 and 10 min, respectively

*SUV*_*max,liver*_ SUV_max_ of liver, *SNR* signal-to-noise ratio, *SUV*_*max,lesion*_ SUV_max_ of lesion, *TLR* lesion-to-liver ratio

#### Subjective assessment of image quality

Table 5 shows the overall subjective image quality scores, and the inter-rater agreement of subjective image quality was excellent with a kappa of 0.851 (0.829–0.874). The average subjective score was highest for G192-3 (4.99 ± 0.06) and G192-5(4.99 ± 0.03), which is consistent with the SNR. The subjective score increased as the scanning duration increased and the imaging voxel size turned larger. The mean score for G512-3, G512-5, G512-8, G512-10, G1024-8, and G1024-10 were larger than 3, indicating that the image quality of these groups could satisfy clinical diagnostic requirement.

**Table 5:** Subjective image quality scores for the combinations of the different imaging matrices and scanning durations

Group	Subjective score
G192-3	4.99 ± 0.06
G192-5	4.99 ± 0.03
G512-3	3.04 ± 0.18
G512-5	3.57 ± 0.37
G512-8	4.01 ± 0.05
G512-10	4.22 ± 0.36
G1024-3	2.14 ± 0.15
G1024-5	2.97 ± 0.11
G1024-8	3.10 ± 0.10
G1024-10	3.79 ± 0.17

#### Lesion detection

A total of 129 lesions assessed this study [35 (maximum diameter ≤ 7 mm), 76 (7 mm < maximum diameter ≤ 15 mm), and 18 (15 mm < maximum diameter ≤ 25 mm)]. In visual assessment, only one false-positive lesion was found in G512-3, G1024-3, and G1024-5, respectively. The number of false-negative lesions found in G192-3, G192-5, G512-3, G1024-3, and G1024-5 was 5, 4, 1, 4, and 1, respectively. Among the lesions appeared as false negatives, 4 had a maximum diameter of less than 7 mm (with an average size of about 5 mm), and 1 about 15 mm in diameter. All lesions had SUV_max_ between 3.1 and 3.8 in G512-5.

As shown in Table 3, TLR in G192 was lower than that in G512 and G1024. TLR decreased as the scanning duration increased for the same imaging matrix, and the difference was little between G512 and G1024. The mean TLR in G1024-3 was the highest among all the combinations. For the groups without focal misdiagnosis found, G1024-8 had the highest TLR.

### Reconstruction time and storage space occupation

Based on the current data reconstruction equipment in our department, the reconstruction time and storage space occupation for each sequence with different matrices and scanning durations combinations are listed in Table 6.

**Table 6:** Reconstruction time and storage occupation of ^18^F-FDG PET/CT images with different matrices

Voxel size (mm^3^)	Mean storage occupied per slice in 2D (kB)	Reconstruction time of Image sequence (min)
3.1 × 3.1 × 1.4	76.47	5
1.2 × 1.2 × 1.4	516.57	12
0.6 × 0.6 × 1.4	2051.53	40

The reconstruction time and the storage space occupation for the same matrix with different scanning durations were almost the same; therefore, only the reconstruction time for different matrices was listed. As shown in this table, the image reconstruction time increased significantly as the matrix increased, and the storage space occupied was roughly proportional related to the square of the matrix. The reconstruction time for G1024 is about three–four times longer than that for G512 and about eight times longer than that for G192.

## Discussion

Limited by the amount of the injected dose, collecting more counts can be achieved only by increasing the scan time in the conventional PET/CT, which may cause possible motion artifacts and less clinical examination circulation. Taking the advantage of the high sensitivity of uEXPLORER PET/CT, we have evaluated the different combinations of imaging voxel sizes and scanning duration based on both phantom and clinical studies and found that small voxel imaging at the millimeter or submillimeter level can improve lesion detection capabilities.

The accurate evaluation of metastatic colorectal cancer (mCRC) [21, 22] is directly related to the choice of treatment and the judgment of prognosis [23]. In this study, we choose the colorectal cancer for our clinical evaluation because mCRC is prone to relatively fixed-position small-size, hypometabolic, and irregular metastatic. Using a long-axis PET/CT with small voxel imaging can help detect and evaluate such lesions was clinically significant. Due to the inaccurate boundary outlining by the threshold method in some small lesions and artifacts by the manual outlining in the individual sequences of a patient, the metabolic tumor volume (MTV) and total lesion glycolysis (TLG) were not included in this study [24].

Yan et.al pointed out that the square of SNR in PET is proportional to the product of the system sensitivity, the injected radioactivity, and the scanning duration [25]. Hu et al. proved that uEXPLORER PET/CT imaging at a dose of 3.7 MBq/kg with 2 min scanning duration for 192 by 192 matrix reconstruction can achieve clinically usable image quality [12]. In the pre-evaluation, the SNR in G192-3 is 1.6 and 2.0 times higher than that of G512-3 and G1024-3, respectively, as shown in Table 3. Following this, the scanning duration for G512 and G1024 should be about 2.5 times and four times longer than that of G192, and we set up the individual reconstruction combinations in this study.

The results in this study have demonstrated that small voxel PET/CT imaging can improve SUV_max_, which is consistent with the previous results reported by several studies [24, 26]. This is because the high metabolism is less likely to be averaged, which can result in an increase in SUV_max_. Furthermore, the SUV_max_ reveals the amount of decays measured at the level of individual pixels/voxel, which is more sensitive to noise. If not enough data is collected per voxel, SUV_max_ will be biased and decreases till it reaches a stable state as increasing the scanning duration. Therefore, physicians should select higher cutoff values required for small voxel imaging [11]. On the other hand, SUV_mean_ is not as sensitive to noise as SUV_max_ due to the averaged statistical fluctuation when calculating the mean value, which accounted for the little difference in SUV_mean_ among most groups as shown in Table 3. These relationships have been demonstrated in both the phantom and clinical studies. As a metric for evaluating the noise of images, SD could affect SNR together with SUV_mean_.

In the comparison among (1) 5 min, 8 min, and 10 min groups of G512 and (2) 8 min and 10 min groups of G1024, significant differences in SUV_max_, SD, SNR, and SUV_max, lesion_ but little difference in SUV_mean_, TLR, and lesion detection accuracy were identified. G512-5 and G1024-8 can achieve a similar result in SUV_max_, SUV_mean_, SD, SNR, TLR, and SUV_max,lesion_.

**Fig. 5:**
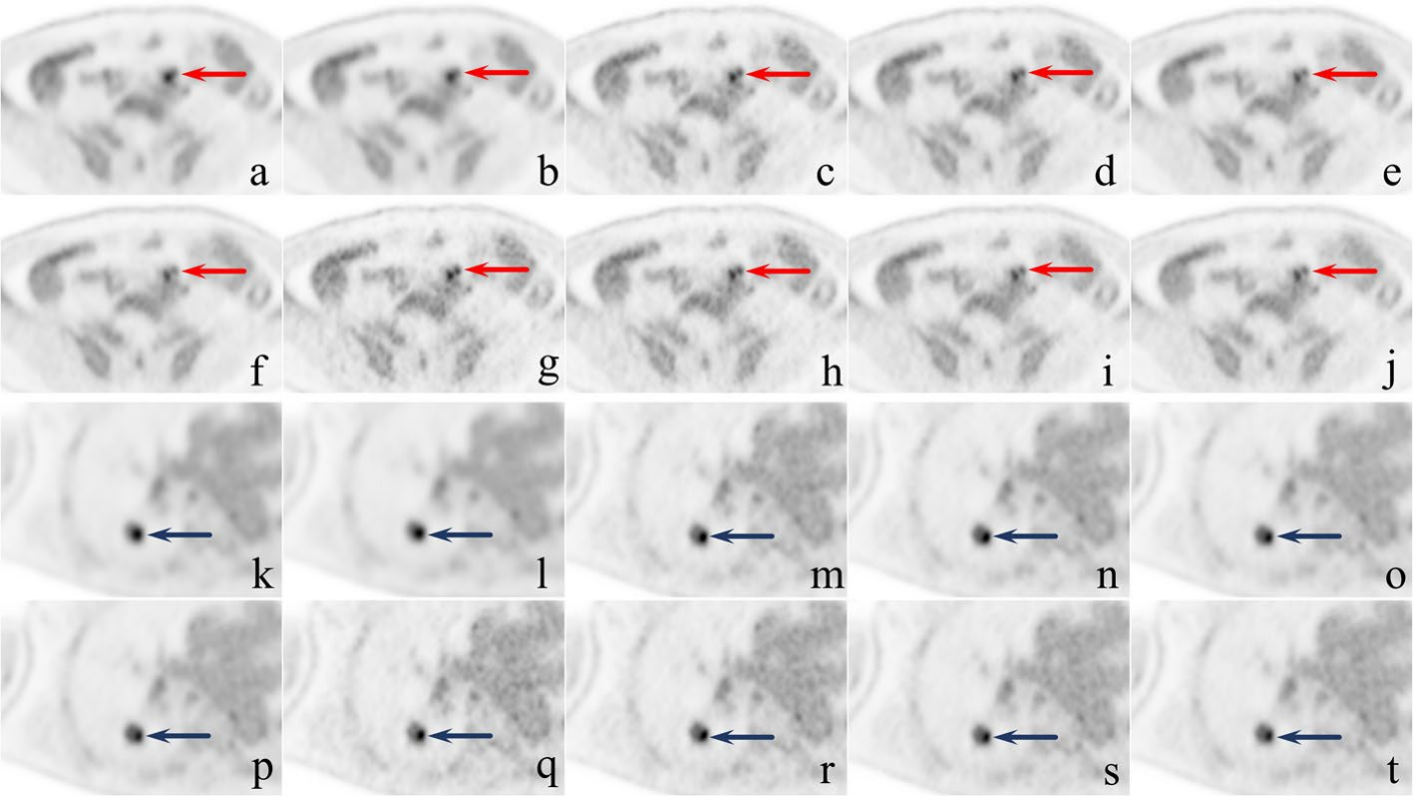
The value of large matrices in differentiating adjacent lesions and evaluating the internal details of lesions. A 62-year-old male was diagnosed with rectum adenocarcinoma with parenteral lymph node metastasis (**a**–**j**). The metastatic lymph nodes are visible in all sub-figures (pointed by red arrows) in front of the sacrum. On the plot of G192 (**a**, **b**), only one hot area with irregular morphology can be seen, but the two adjacent metastatic lymph nodes can be clearly distinguished on G512 (**C**–**F**) and G1024 (**G**–**J**). A 63-year-old female was diagnosed with pulmonary metastases from adenocarcinoma of the sigmoid colon (**k**–**t**). Pulmonary metastases can be clearly seen on all sub-figures (pointed by blue arrows). It could be seen as one homogeneous hot area on the G192 (**k**–**l**), but on G512 and G1024, the hypermetabolic zone can be seen mainly in the inner posterior side of the mass

The key benefit introduced by smaller voxel imaging is a higher spatial resolution, which could improve to differentiate adjacent lesions pointed by red arrows as shown in sub-figure a–j in Fig. 5. The smaller imaging size alleviates PVE and makes the boundary of small lesions clearer. Besides, the higher spatial resolution also allows us to evaluate the metabolic distribution within the lesion more precisely, as pointed by blue arrows in sub-figure k–t in Fig. 5, which can bring additional benefits to the evaluation of disease details or efficacy evaluation.

**Fig. 6:**
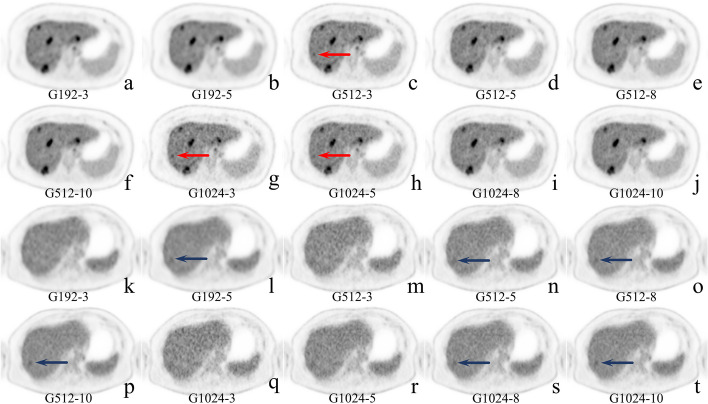
Typical cases with false-positive and false-negative lesions. A 35-year-old female was diagnosed with liver metastases from rectal cancer (**a**–**j**). G512-3 (**c**), G1024-3 (**g**), and G1024-5 (**h**) showed a positive lesion that was not present on images from longer acquisition times and MR images. It was identified as a false positive. A 58-year-old male was diagnosed with adenocarcinoma of the colon with multiple metastases to the liver (**k**–**t**). G192-5 (**c**), G512-5 (**n**), G512-8 (**o**), G512-10 (**p**), G1024-8 (**s**), and G1024-10(t) showed a positive lesion in the right lobe of the liver(pointed by blue arrows), which could not be seen in the rest of sub-figures. It was identified as a false negative in G192-3 (**k**), G512-3 (**m**), G1024-3 (**q**), and G1024-5 (**r**)

Small voxel imaging had a significantly higher TLR, which means a higher lesion detection ability [27]. The TLR increased with the increase of the matrix or the shortening of the acquisition time. However, the noise was also amplified in this process, which may hide some small or hypometabolic lesions. It is important to note that the high image quality also needs to balance TLR and the false-positive rate or false-negative in clinical use. Figure 6 shows a representative false-positive lesion and a representative false-negative lesion.

In total, a large reconstruction imaging matrix can improve the spatial resolution, alleviate PVE, and enhance the small lesion detection ability at the expense of the image SNR and contrast due to fewer data measured in each voxel. This is why the protocols in low dose and fast PET acquisition are not suitable for small voxel imaging [10].

From the results of this study, we concluded that the following groups: G512-5, G512-8, G512-10, G1024-8, and G1024-10 can achieve smaller SD, better SNR, and superior diagnostic accuracy, which could be suggested for clinical practice.

The reconstruction time and storage space occupation were significantly higher for the 1024 matrix. Considering all the factors, the matrix of 512 × 512 with a scanning duration of 5 min is more suitable for the clinical applications.

Matrix of 1024 × 1024 could achieve a higher TLR but has little statistically significant difference in most groups with different scanning durations, which showed the potential value in detecting small or hypometabolic lesions, and left as the future work.

This study has several limitations. Firstly, this was a single-center retrospective study, with a small number of cases, and the results might be influenced by selection bias. Secondly, artificial intelligence algorithms for further improving image quality were not included in this work, which could be evaluated in future studies.

## Conclusions

This exploratory study found that PET/CT imaging with small voxel can improve SUV_max_, and TLR of lesions, which is advantageous for the diagnosis of small or hypometabolic lesions if with sufficient counts. With an ^18^F-FDG injection dose of 3.7 MBq/kg, uEXPLORER PET/CT imaging with a matrix size of 512 × 512 with 5 min or 1024 × 1024 with 8 min can obtain images that met the requirements for clinical use.

### Supplementary Information

**Additional file 1**. Objective evaluation indexes and comparative results of mediastinal and gluteus maximus image quality at different imaging conditions

## Data Availability

The data that support the findings of this study are available from the corresponding author upon reasonable request.
