# Supplementary material for: Phantom study and clinical application of total-body 18F-FDG PET/CT imaging: How to use small voxel imaging better?
Source: EJNMMI Phys. 2024 Feb 15;11:17. doi: 10.1186/s40658-023-00597-w (PMC10869323; doi:10.1186/s40658-023-00597-w)
Supplement: Supplementary file 1 — Additional file 1. Objective evaluation indexes and comparative results of mediastinal and gluteus maximus image quality at different imaging conditions [file 40658_2023_597_MOESM1_ESM.docx]

Supplementary table 1 The objective evaluation indexes at mediastinum and gluteus maximus for the combinations of the different imaging matrices and scanning durations

| Index | G192-3 | G192-5 | G512-3 | G512-5 | G512-8 | G512-10 | G1024-3 | G1024-5 | G1024-8 | G1024-10 |
| --- | --- | --- | --- | --- | --- | --- | --- | --- | --- | --- |
| SUV_max,med_ | 2.01±0.29 | 2.00±0.29 | 2.15±0.31 | 2.10±0.30 | 2.04±0.29 | 2.01±0.28 | 2.35±0.34 | 2.25±0.31 | 2.13±0.30 | 2.11±0.29 |
| SUV_mean,med_ | 1.77±0.29 | 1.76±0.28 | 1.76±0.28 | 1.75±0.28 | 1.72±0.27 | 1.71±0.27 | 1.76±0.28 | 1.75±0.28 | 1.73±0.27 | 1.72±0.26 |
| SD_med_ | 0.11±0.02 | 0.10±0.02 | 0.15±0.02 | 0.13±0.02 | 0.11±0.02 | 0.10±0.02 | 0.21±0.04 | 0.16±0.03 | 0.14±0.02 | 0.13±0.02 |
| SNR_med_ | 17.34±4.01 | 18.59±5.41 | 11.81±2.18 | 13.99±2.94 | 16.08±3.31 | 16.85±3.67 | 8.73±1.82 | 10.83±2.09 | 12.61±2.04 | 13.59±2.37 |
| SUV_max,GM_ | 0.71±0.13 | 0.71±0.12 | 0.80±0.14 | 0.78±0.14 | 0.76±0.13 | 0.75±0.13 | 0.87±0.15 | 0.83±0.14 | 0.80±0.13 | 0.78±0.13 |
| SUV_mean,GM_ | 0.62±0.11 | 0.62±0.11 | 0.61±0.11 | 0.62±0.11 | 0.62±0.11 | 0.62±0.11 | 0.62±0.11 | 0.62±0.11 | 0.62±0.11 | 0.62±0.11 |
| SD_GM_ | 0.05±0.01 | 0.04±0.01 | 0.07±0.02 | 0.06±0.02 | 0.06±0.02 | 0.05±0.02 | 0.10±0.02 | 0.08±0.01 | 0.07±0.01 | 0.06±0.01 |
| SNR_GM_ | 14.61±3.75 | 15.69±3.51 | 8.52±1.46 | 10.07±1.66 | 11.03±2.37 | 11.84±2.47 | 6.54±0.98 | 7.78±1.18 | 9.25±1.44 | 10.05±1.74 |

SUV_max,med_, SUV_max_ of mediastinal blood pool；SUV_max,GM_, SUV_max_ of gluteus maximus

Supplementary table 2 Comparisons of objective evaluation indexes of image quality at mediastinal and gluteus maximus among different scan time within imaging matrix.

| Matrix | Index | *P_0_* | *P_1_* | *P_2_* | *P_3_* | *P_4_* | *P_5_* | *P_6_* |
| --- | --- | --- | --- | --- | --- | --- | --- | --- |
| 512 | SUV_max,med_ | <0.001 | 0.073 | <0.001 | <0.001 | 0.007 | 0.001 | 0.194 |
|  | SUV_mean,med_ | <0.001 | 0.528 | 0.001 | <0.001 | 0.001 | <0.001 | 0.006 |
|  | SD_med_ | <0.001 | <0.001 | <0.001 | <0.001 | <0.001 | <0.001 | 0.262 |
|  | SNR_med_ | <0.001 | <0.001 | <0.001 | <0.001 | <0.001 | <0.001 | 0.104 |
| 1024 | SUV_max,med_ | <0.001 | 0.004 | <0.001 | <0.001 | <0.001 | <0.001 | 0.537 |
|  | SUV_mean,med_ | <0.001 | 0.870 | 0.007 | 0.005 | 0.020 | 0.004 | 0.010 |
|  | SD_med_ | <0.001 | <0.001 | <0.001 | <0.001 | <0.001 | <0.001 | 0.016 |
|  | SNR_med_ | <0.001 | <0.001 | <0.001 | <0.001 | <0.001 | <0.001 | 0.021 |
| 512 | SUV_max,GM_ | <0.001 | 0.011 | 0.001 | <0.001 | 0.018 | 0.001 | 0.334 |
|  | SUV_mean,GM_ | 0.358 | 1.000 | 0.325 | 0.175 | 1.000 | 0.848 | 1.000 |
|  | SD_GM_ | <0.001 | <0.001 | <0.001 | <0.001 | 0.141 | 0.001 | 0.001 |
|  | SNR_GM_ | <0.001 | <0.001 | <0.001 | <0.001 | 0.026 | <0.001 | 0.002 |
| 1024 | SUV_max,GM_ | <0.001 | 0.001 | <0.001 | <0.001 | 0.002 | <0.001 | 0.143 |
|  | SUV_mean,GM_ | 0.312 | 1.000 | 0.354 | 0.655 | 0.200 | 0.409 | 1.000 |
|  | SD_GM_ | <0.001 | <0.001 | <0.001 | <0.001 | <0.001 | <0.001 | <0.001 |
|  | SNR_GM_ | <0.001 | <0.001 | <0.001 | <0.001 | <0.001 | <0.001 | <0.001 |

**Note:** *P_0_* indicates *P* values of Freidman test among four time groups; *P_1_* to *P_6_* indicates adjusted *P* values after Bonferroni corrections from comparisons between 3min and 5min, between 3min and 8min, between 3min and 10min, between 5min and 8min, between 5min and 10min, and between 8min and 10min, respectively.
